# A study to evaluate the effectiveness of Best Beginnings’ Baby Buddy phone app in England: a protocol paper

**DOI:** 10.1017/S1463423618000294

**Published:** 2018-07-23

**Authors:** Toity Deave, Sally Kendal, Raghu Lingam, Crispin Day, Trudy Goodenough, Elizabeth Bailey, Sam Ginja, Sam Nightingale, Jane Coad

**Affiliations:** 1 Centre for Child & Adolescent Health, University of the West of England, Faculty of Health & Applied Sciences, Oakfield House, Oakfield Grove, Bristol, UK; 2 Centre for Health Services Studies, University of Kent, Canterbury, UK; 3 School of Women’s & Children’s Health, University of New South Wales, Australia; 4 Child & Adolescent Mental Health Service Research Unit, Guy’s Munro Centre, London, UK; 5 Centre for Technology Enabled Health Research, Coventry University, Coventry, UK; 6 Institute of Health & Society, Newcastle University, Newcastle upon Tyne, UK

**Keywords:** app, Best Beginnings, evaluation, parenting, pregnancy

## Abstract

**Introduction:**

Developments in information and communication technologies have enabled electronic health and seen a huge expansion over the last decade. This has increased the possibility of self-management of health issues.

**Purpose:**

To assess the effectiveness of the Baby Buddy app on maternal self-efficacy and mental well-being three months post-birth in a sample of mothers recruited antenatally. In addition, to explore when, why and how mothers use the app and consider any benefits the app may offer them in relation to their parenting, health, relationships or communication with their child, friends, family members or health professionals.

**Methods:**

We will use a mixed-methods approach, a cohort study, a qualitative element and analysis of in-app data. Participants will be first-time pregnant women, aged 16 years and over, between 12 and 16 weeks of gestation and recruited from five English study sites.

**Evaluation plan:**

We will compare maternal self-efficacy and mental health at three months post-delivery in mothers who have downloaded the Baby Buddy app compared with those that have not downloaded the app, controlling for confounding factors. Women will be recruited antenatally between 12 and 16 weeks of gestation. Further follow-ups will take place at 35 weeks of gestation and three months post-birth. Data from the cohort study will be supplemented by in-app data that will include, for example, patterns of usage. Qualitative data will assess the impact of the app on the lives of pregnant women and health professionals using both focus groups and interviews.

**Ethics:**

Approval from the West Midlands-South Birmingham Research Ethics Committee (NRES) (16/WM/0029) and the University of the West of England, Bristol, Research Ethics Committee (HAS.16.08.001).

**Dissemination:**

Findings of the study will be published in peer reviewed and professional journals, presented locally, nationally and at international conferences. Participants will receive a summary of the findings and the results will be published on Best Beginnings’ website.

## Introduction

There has been a huge increase in the last 10 years of electronic health (e-Health) and mobile health (m-Health) solutions to increase self-management of health problems in high, medium and low-income countries (Goetz *et al*., 2017). This has been made possible by developments in information and communication technologies. Mobile phones have capabilities that were, previously, only available by using computers and portable devices, such as laptops. Access to the internet is now widespread and this has underpinned the development of ‘applications’ or apps; these have led to a fundamental change in the methods that people use to access information. Apps offer wider opportunities for the provision of healthcare and information than have ever been known before. This has also brought about changes in the demands that patients make of healthcare services (Hussain *et al*., 2015).

The population of app users includes expectant and new mothers with whom internet-based information about pregnancy has been popular for nearly a decade (Bernhardt and Felter, 2004; Declercq *et al*., 2013). In 2013, around 1500 pregnancy apps were available on the Google Play app store and the Apple iStore (Tripp *et al*., 2014) and there are likely to be many more if this search was repeated. Whilst e-Health and m-Health have the potential for enhancing traditionally delivered healthcare services (Tripp *et al*., 2014; Lau *et al*., 2016), little is known about the use and effectiveness of pregnancy- or perinatal-related smartphone apps (Rodger *et al*., 2013). A systematic review of apps for women’s health concluded that more research was needed, especially in relation to the use and embedding of apps in healthcare settings (Derbyshire and Dancey, 2013). Most expectant women access the internet daily (Waring *et al*., 2014) and pregnant women who were asked about using the internet for searching for information perceived the information that they accessed to be reliable but they had not discussed them with their midwife (Larsson, 2009). The most popular apps by consumer rating were those that were interactive (Tripp *et al*., 2014).

The Baby Buddy app was launched in England in November 2014. It was developed by the national child health and well-being charity, ‘Best Beginnings’, who describes the app as ‘an electronically delivered health intervention’ that is intended to support and guide women through pregnancy (antenatal side of the app) and the first six months of their child’s life (postnatal side of the app). Best Beginnings designed the app to inform and empower women of all ages and from wide socio-demographic backgrounds so that all expectant parents could search for and benefit from high quality information and support during pregnancy. Many women may access information online that is unreliable (Rodger *et al*., 2013) but Best Beginnings worked closely with health professionals to ensure that Baby Buddy provides evidence-based, trustworthy information (https://www.bestbeginnings.org.uk/vision). It provides interactive information to help parents look after their own physical and mental health during pregnancy and early parenthood and support them with caring for the physical and emotional health and well-being of their child. It was designed to complement maternity and postnatal services. The content of the app is written so that is can be understood by anyone with a reading age of 11 years or above with a ‘read aloud’ element available. It can be downloaded free of charge from the Apple iStore and the Google Play app store by anyone with a suitable smartphone, including fathers and other family members or friends.

The Baby Buddy app has been developed specifically to target expectant mothers who are under 25 years old but also be attractive to wide range of mothers. Young (teenage) mothers in the United Kingdom are less likely to both engage with formal maternity service provision early in their pregnancy and attend their appointments (Department of Health, 2009; Bradshaw *et al*., 2014); these are risk factors for maternal and infant mortality (Knight *et al.* 2017).

The BaBBLeS study (Bumps and BaBies Longitudinal Study) aims to assess the effectiveness of the Baby Buddy app on improving maternal self-efficacy and mental well-being. It also aims to understand when, why and how mothers use the app and any benefits the app may offer them in relation to their parenting, health, relationships or communication with their child, friends and family members or health professionals.

## Objectives

1. To assess the effectiveness of the Baby Buddy app, at three months post-delivery on maternal self-efficacy and well-being in those mothers who used the Baby Buddy app compared with those who did not use the app;
2. To assess the effectiveness of the Baby Buddy app, at three months post-delivery, on maternal self-efficacy and well-being in those mothers who were shown how to use the app by a health professional compared to those who were not shown or did not download it;
3. To explore how the app has affected the day-to-day lives of participant mothers, specifically around their self-efficacy, parenting ability, health behaviour, interactions and communications with their friends, family and health professionals;
4. To obtain in-depth information from health professionals around their awareness of the Baby Buddy app and barriers and facilitators to integration of the app into routine healthcare.
5. To describe data on the uptake, patterns of usage and detailed analytics of key factors within the app.

## Methodology

This longitudinal study will be conducted, in parallel, in five geographical sites in England and will employ a mixed-methods approach. These areas have been chosen as they are geographically, ethnically and socio-economically diverse and are areas where the app was reported to be well embedded.

This study has three component parts that address the stated objectives: a cohort study, a qualitative element and analysis of in-app data. The cohort study will compare the self-reported self-efficacy and mental well-being of mothers three months post-delivery who downloaded the Baby Buddy app compared with those who did not download the app. The study will control for baseline characteristics, including demographic characteristics, use of technology, social support and baseline levels of self-efficacy and mental well-being. The qualitative element will include mothers and professionals taking part in one–one interviews or focus groups. The in-app data on the uptake, patterns of usage and detailed analytics of key factors within the app will be collected from downloads provided by Best Beginnings (see below).

## Recruitment

Recruitment will take place between June and October 2016. Women who aged 16 years and over, with no previous live child and between 12 and 16 weeks and six days of gestation will be identified from the maternity units in each of the five study sites. Maternity unit administrative clerks, with support of the research midwives in each of the five geographical areas will be asked to undertake a database search for women who fulfil the inclusion criteria. Each woman who is identified will be sent or given a participant information booklet combining the study invitation letter and information booklet by the midwifery services staff and opportunities to discuss it by telephone, text or e-mail. Women in three of the sites will also be invited to take part in further, nested qualitative studies. Mothers can choose to complete the baseline questionnaire online or on paper and will be offered a £5 thank you voucher. A two-week reminder will be sent to each woman who has not responded.

Midwives and health visitors who work in three study sites will be invited to take part in a focus group between September 2016 and February 2017. If more than 10 health professionals agree to take part in each site purposive sampling will be employed to ensure that all geographical areas are covered.

Mothers and health professionals will be informed they can withdraw from the project at their own request. The mothers will be made aware that it would not affect their future care or support from the health professionals.

### Data collection

#### Quantitative data

Baseline data will be collected at the same time as women agree to participate in the study through completion of a questionnaire. Baseline and follow-up data are being collected by self-completion questionnaire from the mothers. The baseline questionnaire asks questions about the mothers’ socio-demographic details, validated tools to measure parenting self-efficacy, anticipated feeding practices, pregnancy dates (expected date of delivery) use of the internet, apps and social media, sources of information about pregnancy and motherhood and social support.

Two follow-up questionnaires will be administered with reminders, using methods outlined above, at 35 weeks of pregnancy and at three months post-birth. The 35-week questionnaire will repeat the validated tools with a postal reminder. At three months post-birth, the questionnaire will repeat the validated tools and will also include questions relating to app usage, health service use, interaction with health services, childbirth experience; the initial reminder will be by telephone. Quantitative data will be entered onto one database at the University of the West of England, Bristol and stored on the university server.

#### Qualitative data

For those women who agree to take part in the qualitative study (as part of the recruitment process in the three selected evaluation sites), they will be invited to take part in in-depth semi-structured interviews and/or a focus group (up to 10 interviews per three geographical sites and to attend with their partner/chosen friend. The interviews will be offered both via the telephone or face-to-face in each geographical area as per individual preference. Their data will be recorded digitally and transcribed verbatim. Data from the one-to-one interviews will be structured in a similar way as the focus group discussions to ensure congruence of data.

For the focus groups, an appreciative inquiry approach will be used with prompts, as developed by the research team and advisors. This element will aim to explore when, why and how mothers use the app and the perceived benefits the app gives them in relation to their parenting, health, relationships and communication with their child, friends and other family members. It will specifically explore how the app is used by mothers to enhance communication with healthcare providers to make ‘every contact count’ (NHS, 2015) and the reasons behind differences in rates of usage, including what makes the app enjoyable to use and responses to embedding activities.

One–one interviews and focus groups will take place between three and six months post-delivery; focus groups will be ‘baby friendly’ to accommodate additional needs.

#### Health professionals

Health professionals’ awareness of the Baby Buddy app and the barriers and facilitators to the integration of the app to usual service delivery will be collected.

Midwives and health visitors in the selected three study sites will be invited to take part in the study; the sample will be up to 10 professionals in each area (*n*=30). Their views will also be sought to explore any ‘added value’ of the apps in supporting mothers, partners/supporters and their babies. Either face-to-face or telephone interviews and/or focus groups will be offered to professionals in each chosen geographical area between April 2017 and October 2017. Their data will be recorded digitally and transcribed verbatim. They will be encouraged to contribute to a shared, integrated perspective in evaluating the app of ‘what works well’ and ‘what could work better’ in integrating the use of the app into healthcare encounters. This is an important aspect of the study, in which the process of using the app will be explored. We will also determine preference/choice through an action learning set approach.

#### In-app data

At the 35-week gestation data collection, mothers will receive an information sheet and consent form to take part in this element of the study. The majority of the patterns of Baby Buddy app use are recorded and stored on secured databases hosted by Best Beginnings as part of a standard procedure necessary for managing and debugging the app. Best Beginnings will provide the research team with limited and secured download access to the database with the ability to obtain specific in-app data from the users of the app. These specific data will include information such as duration of sessions using the app, count of these sessions, flow of app use and general user information. The obtained data will be downloaded and stored in the encrypted format on highly secured university computers. Once all data collection is complete, relevant data will be merged onto one database before analysis but with an appropriate encoding of personal information (ie, anonymised ID codes, not e-mail addresses or postcodes) in order to preserve high level of user confidentiality and anonymity.

### Outcome measures

One main aim for the app is that it increases mothers’ confidence and self-efficacy with regard to pregnancy, baby-care and early parenthood. Therefore, our primary outcome measure was chosen to reflect this, the Tool to measure Parenting Self-Efficacy (TOPSE) (Kendall and Bloomfield, 2005; Bloomfield and Kendall, 2007). The theoretical underpinning of TOPSE is based on the self-efficacy theory developed by Albert Bandura (Bandura, 1982; 1986; 1989). TOPSE measures change in self-efficacy between time-points and is therefore ideally placed to measure outcomes in this study. The 0–6-month version of TOPSE will be adapted, with the author’s help, to measure parenting self-efficacy expectations during pregnancy. TOPSE is a multidimensional instrument of 48 statements within eight scales, each scale having six statements and representing a distinct dimension of parenting: emotion and affection, play and enjoyment, empathy and understanding, control, discipline and boundaries, pressures, self-acceptance, learning and knowledge. The items are rated on an 11-point Likert scale where 0 represents completely disagree and 10 represents completely agree. The scale contains positive and negatively worded items and the responses are summed to create a total score; the lower the score, the lower the level of parenting self-efficacy. Internal reliability coefficients for the subscales ranged from 0.80 to 0.89 and the overall scale reliability was 0.94. External reliability coefficients ranged from *r*_s_=0.58 (*n*=19, *P*<0.01) to *r*_s_=0.88 (*n*=19, *P*<0.01).

The secondary outcomes are the Warwick-Edinburgh Mental Well-Being Scale (WEMWBS) and the in-app data. The WEMWBS is a 14-item scale of mental well-being covering subjective well-being and psychological functioning, in which all items are worded positively and address aspects of positive mental health (Tennant *et al*., 2007). The scale is scored by summing responses to each item answered on a 1–5 Likert scale. The minimum scale score is 14 and the maximum is 70. WEMWBS has been validated for use in the United Kingdom with those aged 16 and above. From the in-app data, the uptake, patterns of usage and detailed analytics of key factors within the app will be will be analysed (see below). This scale comprises of 14 statements describing feelings (eg,‘I have been feeling useful’) and functional aspects (eg, ‘I’ve been dealing with problems well’) over the previous two weeks. Items were scored from 1 (none of the time) to 5 (all of the time) and summed to provide an overall score between 14 and 70, where higher scores corresponded to greater frequency. WEMWBS has demonstrated good content and criterion-related validity and high test–retest reliability (0.83) when used in different samples and public health contexts, including parenting programmes (Stewart-Brown *et al*., 2011).

Additional data to be collected and measures to be used include the mother’s socio-demographic factors (age, ethnic group, highest level of education attained, relationship status and employment), intention and actual breastfeeding, the multidimensional scale of perceived social support (Zimet *et al*., 1988) and use of social media and technologies, adapted from the Media and Technology Usage and Attitudes Scale (Rosen *et al*., 2013). In both the second and final questionnaires, mothers will be asked whether they had downloaded and/or used the Baby Buddy app and how they had heard about it (source of information). In the final, three month post-delivery questionnaire, the Childbirth Experience Questionnaire (Walker *et al*, 2015) is included.

### Sample sizes

#### Quantitative

The average percentage of app download across our five sites is 13.6%, based on data provided by Best Beginnings. Assuming that the percentage of mothers who downloaded the app is slightly below that rate, at 12.5% and assuming a 30% loss to follow-up, the study will need to recruit 559 women into the study to have 90% power to detect a 0.5 SD difference in the overall TOPSE (primary outcome) across domains. If the proportion of mothers in the final sample who downloaded the app changes then the required sample size will change accordingly.

#### Qualitative

The sample sizes for the qualitative study are based on an estimate to expect saturation of data on themes emerging from the study. A maximum of 30 in-depth interviews (*n*=30, 10 from each of the selected three sites will be conducted with women who have used the app and who have consented to participate. There will be three focus groups (*n*=30 mothers), one group per three sites with 10 attendees in each group. A balanced group of recruits to interviews and focus groups will be spread across the three sites to allow for local differences in experience with the app use and professional encounters.

The same rationale is used for health professionals’ views (*n*=30, 10 from each of three sites). The invitation to health professionals will be to attend a focus group with the option of telephone interview if unable to attend groups and to ensure the target sample size is achieved. This will facilitate reaching data saturation against the backdrop of ‘real world’ demands on professionals’ time and availability.

### Data analysis

#### Quantitative data

Descriptive statistics will be used to describe the sample, including the mothers’ age, socio-demographic, ethnicity, access to and use of technology and the overall scores for the outcome measures. Linear regression models will be used to compare primary and secondary outcomes in mothers who used the app compared with those who did not use the app, depending on outcome data distribution. If outcome data are not normally distribution, the possibility of logistic regression analysis will be explored, using binary outcomes. Key baseline demographic variables will be used to control for potential confounding variables, including maternal age, education, employment, relationship status, recruitment site, social support, general technology use and use of other pregnancy apps. Baseline levels of the outcome variables, for example, maternal self-efficacy, will be controlled for in the final analysis. Analysis will be as per protocol and analysis plan. Regression methods will control for baseline demographic characteristics. All analyses will be carried out using Stata software. Missing data will not be accounted for using multiple imputation, unless 30% of the data or more is missing.

A second analysis will compare primary and secondary outcomes, as stated above, between those mothers who heard about the app from a health professional (embedded use) and those women who did not hear about it or who did not download the app by three months post-delivery. The above statistical methods will be used again to compare outcomes between ‘download and heard about the app from healthcare professional’ and ‘downloaded but did not hear about the app from a healthcare professional, or did not download it’.

#### Qualitative data

Divergence and concordance of opinion will be explored in the data from the interviews and focus groups.

#### In-app data

Data orientation will be undertaken and then formatted for analysis. This will include an exploratory analysis of the basic socio-demographic information and profiling of app users (eg, age, occupation, education, ethnic origin); a description of the patterns of app use: the creation of the avatar; uses of the goal setting function, ‘ask me a question’; ‘what does that mean’; the media downloaded; a detailed analysis of interactions between various factors (eg, age by frequency of use, location by number of downloads).

The findings from each section of the study will be discussed with the user group and their views of interpretation from their perspectives will be sought. A synthesis of all the information collected will be collated to form the final report.

### Anonymity and data protection

All participants are being assigned a unique identity code (UIC) to facilitate the conduct and analysis of the study. These will be used on all paper and electronic versions of questionnaires, interview transcriptions and databases. Recruitment logs will serve as a separate confidential record of the participants’ details. This will permit identification of all participants recruited to the study, in accordance with regulatory requirements and for follow-up, as required. Two logs will be created: one for women participants and one for the health professionals.

In addition, the following data will be collected and stored on a separate database: mother’s UIC, name, address, telephone and other relevant contact details, for example, date follow-up questionnaires due, preferred format for administration of follow-up questionnaires, e-mail address.

For the interview data, with the permission of the interviewees, interviews will be digitally audio-recorded. They will be anonymised, transcribed verbatim and stored with the other study data. The transcribed interviews will be treated as confidential documents and held securely with no identifiable information on the transcripts. These will be stored on a secure password-protected University server.

All data collected will be held securely, in a locked room, or locked cupboard or cabinet. Access to the information will be limited to the study research team. Computer-held data, including study databases, will be held on secure, password-protected University or National Health Service (NHS) servers and will be backed up as a minimum, every 24 h. Personal laptops will not be used. Where University or NHS laptops are used, these will be password-protected and data will be transferred to the University/NHS server as soon as possible. At the end of the study, data from all centres will be archived by the University of the West of England, Bristol (UWE) for a period of seven years or longer, if required, as set out in UWE’s Code of Research Conduct.

### Adverse events

The occurrence of adverse events, as a result of participation in this study, is not anticipated. We believe the risks for participants in this study are minimal. However, should an adverse event occur this will be recorded and reported to the Chief Investigator. Any adverse events will also be reported in the final study report.

### Ethical and regulatory aspects

This study has received a favourable opinion from the NHS Research Ethics Committee (NRES) West Midlands-South Birmingham REC (16/WM/0029), the University of the West of England, Bristol Research Ethics Committee (HAS.16.08.001), and the respective study site’s NHS Research & Development (R&D) departments.

### Strengths and limitations of the study

**Strengths**

- Multi-centre longitudinal study
- Large sample size of over 500 participants to be recruited
- We are using mixed methods to help assess and explore the impact of the Baby Buddy app as well as how the app is being used by women and health professionals
- The primary outcome (TOPSE, parenting self-efficacy) is generic across all domains of parenting over time

**Limitations**

- The antenatal version of the TOPSE has been adapted for this study.

### Publication and dissemination policy

The research team regard active, widespread dissemination of outputs from this research as essential and this will include peer reviewed and professional journals, conference presentations and national and local seminars. The research team members will contribute towards drafting the papers, reporting the findings of the study and will be named authors on those papers, providing they fulfil the Vancouver criteria for authorship. If a member of the research team wishes to analyse and report other findings from the study they can do so on the agreement of the other research team members.
